# Adaptation and validation of the scleroderma skin patient-reported outcome questionnaire into Hungarian

**DOI:** 10.1186/s41687-026-01147-5

**Published:** 2026-07-14

**Authors:** Panna Pytel, Ágnes Ágoston-Szabó, Attila Szabó, Gábor Kumánovics, Gabriella Nagy

**Affiliations:** Medical Centre, Department of Rheumatology, Immunology and Musculoskeletal Rehabilitation Clinic, University of Pecs, Pécs, Hungary

**Keywords:** Systemic sclerosis, Quality of life, SSPRO, Validation, Skin involvement, OMERACT filter

## Abstract

**Introduction:**

Skin involvement in patients with systemic sclerosis (SSc) can significantly impair quality of life, affecting psychological, physical, and social well-being. The Scleroderma Skin Patient-Reported Outcome (SSPRO) questionnaire was developed to assess these dimensions.

**Objective:**

The aim of this study was to adapt and validate the SSPRO questionnaire in Hungarian.

**Patients and methods:**

The study enrolled 60 patients with SSc [53 women, 7 men; mean age 57.7 ± 13.5 years; diffuse cutaneous SSc *n* = 31 (51.7%)] and 31 age- and sex-matched healthy controls (mean age 57.9 ± 14.9 years). The 18-item SSPRO questionnaire was adapted into Hungarian using a standardized forward–backward cross-cultural translation procedure. Psychometric evaluation was performed in accordance with COSMIN recommendations and included assessment of content validity, structural validity, internal consistency, convergent validity, known-groups validity, reproducibility, and scaling properties.

**Results:**

No significant floor or ceiling effects were observed. Internal consistency was acceptable (Cronbach’s α = 0.789). Structural validity analysis using principal component analysis identified a dominant first component explaining approximately 50% of the total variance, suggesting a primarily unidimensional structure with secondary underlying dimensions. Convergent validity analysis demonstrated significant correlations between SSPRO scores and established measures of quality of life and disease impact. Known-groups validity was confirmed by the ability of the questionnaire to distinguish between patients with SSc and healthy controls, while no significant differences were observed between limited and diffuse cutaneous SSc subgroups. Test–retest reliability demonstrated excellent agreement (ICC(2,1) = 0.894, 95% CI: 0.764–0.932).

**Conclusion:**

The Hungarian version of the SSPRO questionnaire is a reliable and valid instrument for assessing skin-related quality of life in patients with SSc. It provides a quick, simple, and clinically useful tool that captures patient-reported aspects of disease burden not reflected by physician-based measures.

**Supplementary Information:**

The online version contains supplementary material available at https://doi.org/10.1186/s41687-026-01147-5.

## Introduction

Systemic sclerosis (SSc) is a rare, chronic autoimmune connective tissue disease characterized by microvascular damage, complex immune system activation, and pathological connective tissue remodeling associated with fibrosis [1, 2]. The clinical presentation is highly heterogeneous; in addition to skin involvement, internal organ manifestations are frequent and carry major prognostic significance [2]. Among visceral complications, progressive interstitial lung fibrosis, pulmonary arterial hypertension (PAH), cardiac and gastrointestinal involvement, and the potentially life-threatening renal crisis rank among the most severe outcomes [2].

Skin involvement is one of the earliest and most characteristic clinical features of SSc, resulting from microcirculatory damage and progressive fibrosis [3]. Clinical manifestations such as Raynaud’s phenomenon, puffy fingers, sclerodactyly, joint contractures, microstomia, digital ulcers, and telangiectasias impair hand function and daily activities and have a substantial psychological and social impact [4–10].

Due to its heterogeneous manifestations, SSc is associated with a marked reduction in quality of life. Skin involvement, in particular, contributes not only to physical disability but also to emotional distress, altered body image, and social limitations [11].

In recent decades, patient-reported outcomes (PROs) have gained increasing importance in the evaluation of chronic diseases, as they capture patients’ subjective experiences that are not fully reflected by physician-based assessments [12–15]. In rheumatologic diseases, including SSc, this patient-centered approach is strongly supported by international initiatives such as OMERACT, which emphasize the relevance of outcomes that reflect patients’ lived experience [16].

Various generic and disease-specific instruments are available for assessing quality of life in systemic sclerosis, including the Health Assessment Questionnaire Disability Index (HAQ-DI) [17], the Scleroderma Health Assessment Questionnaire (sHAQ) [18], and organ-specific tools such as the UCLA Gastrointestinal Tract Instrument [19]. However, these instruments do not specifically focus on skin-related quality of life. Given the complex physical, emotional, and social impact of skin involvement, the development of dedicated tools became necessary, leading to the creation of the Scleroderma Skin Patient-Reported Outcome (SSPRO) questionnaire [20].

The Scleroderma Skin Patient-Reported Outcome (SSPRO) questionnaire was developed by Man et al. to quantify the impact of skin-related symptoms on quality of life in patients with SSc [20].

The SSPRO questionnaire consists of 18 items assessing physical, emotional, and social aspects related to skin involvement. Responses are recorded on a 7-point Likert scale ranging from 0 to 6, where lower scores indicate better quality of life. The total score ranges from 0 to 108 [19]. The questionnaire includes items addressing physical symptoms of the skin (e.g., tightness, pain, dryness) as well as psychological impacts (e.g., frustration, anxiety, reduced self-confidence). In addition, it evaluates social dimensions such as interpersonal relationships, clothing, and daily activities [20].

The SSPRO offers several advantages: it is quick to complete, effectively differentiates between patients with varying disease severity, and is sensitive to clinical changes, making it suitable as an outcome measure in clinical trials [19].

The aim of the present study was to adapt and validate the SSPRO questionnaire into Hungarian, providing a much-needed tool for the comprehensive assessment of quality of life in patients with SSc.

## Patients and methods

### Patients

We consecutively enrolled 60 patients with SSc who were under care at the Department of Rheumatology and Immunology, Medical Center, University of Pécs, and fulfilled the 2013 EULAR/ACR classification criteria [21]. The majority were women (*n* = 53). The proportions of patients with diffuse cutaneous SSc (dcSSc) and limited cutaneous SSc (lcSSc) were nearly equal (lcSSc *n* = 29 [48.3%], dcSSc *n* = 31 [51.7%]). Thirty-one healthy individuals, matched for age and sex, were included as controls. All participants provided written informed consent after receiving verbal and written information. The study was approved by the Hungarian National Ethics Committee (ETT-TUKEB, approval number BM/29196-1/2023) and conducted in accordance with the principles of the Declaration of Helsinki.

Participants completed a questionnaire package at baseline. The package included the Hungarian version of the Scleroderma Skin Patient-Reported Outcome (SSPRO) questionnaire and several reference instruments used as comparators during validation: HAQ-DI [17], Short Form-36 Health Survey (SF-36) [22], Dermatology Life Quality Index (DLQI) [23], Satisfaction With Appearance Scale-Brief form (Brief-SWAP) [24], sHAQ [18], Visual Analogue Scale (VAS) for fatigue [25], and the Skin Thickness and Thinness Questionnaires [26]. Five days later the SSPRO was filled out again for test-retest assessment.

In addition, detailed clinically relevant data were collected, including medical history, physical examination, laboratory tests (autoantibodies and routine parameters) [27], echocardiography [28], telangiectasia score [29], modified Rodnan Skin Score (mRSS) [30].

Internal organ involvement was defined according to the following criteria:

Interstitial lung disease (ILD) was assessed by two experienced radiologists based on high-resolution computed tomography (HRCT) [31]. PAH was diagnosed if right heart catheterization (RHC) revealed a mean pulmonary arterial pressure > 20 mmHg [32]. Cardiac involvement was considered present in cases of congestive heart failure, pericardial disease, arrhythmia, or diastolic dysfunction [33]. Esophageal involvement was diagnosed if barium swallow test demonstrated stricture or motility disorder [34]. Gastrointestinal involvement was evaluated using the University of California Los Angeles Scleroderma Clinical Trials Consortium Gastrointestinal Tract (UCLA GIT) 2.0 questionnaire [19].

Early disease was defined as ≤ 4 years from the first non-Raynaud symptom, consistent with published definitions [35].

### Statistical methods

Psychometric evaluation was conducted in accordance with COSMIN recommendations and included assessment of content validity (during the translation phase), structural validity, internal consistency, convergent validity, known-groups validity, reproducibility and scaling properties [36]. The questionnaire was adapted using a standardized forward-backward translation procedure with the involvement of an expert committee, following established guidelines for cross-cultural adaptation of self-report measures [37].

Structural validity was explored using principal component analysis. Known-groups validity was assessed using the Mann-Whitney U test to compare SSPRO scores between predefined groups. Correlations were examined using Spearman’s rank correlation. All analyses were performed using IBM SPSS Statistical Data Editor, version 29.0.2.0.

### Adaptation and validation into Hungarian

The original English 18-item questionnaire was adapted into Hungarian using a forward-backward translation process with cultural and linguistic adaptation. Content validity was ensured during the translation process through expert committee review and patient involvement, focusing on item relevance, comprehensibility, and cultural appropriateness.

An initial translation into Hungarian was performed independently by a physician, a healthcare professional, and a patient. Based on these versions, an expert panel (two physicians, one healthcare professional, and one patient with SSc) finalized the Hungarian questionnaire based on consensus. Subsequently, a language teacher and a Hungarian native healthcare professional residing in an English-speaking country back-translated the questionnaire into English. Comparison of the original and back-translated versions revealed no substantial discrepancies, allowing the expert panel to approve the final Hungarian version of the SSPRO questionnaire. The finalized Hungarian version of the SSPRO questionnaire is provided in Appendix A. The finalized Hungarian version was pilot-tested in five patients with SSc to assess clarity, comprehensibility, and usability. The mean SSPRO score in the pilot test was 24, and the average completion time was approximately 3 min. No item required modification based on patient feedback.

## Results

### Clinical characteristics of patients

The mean age of patients was 58.1 ± 13.4 years, and the mean disease duration was 11.7 ± 7.0 years.

Gastrointestinal involvement was present in 31 patients (51.7%). Lung fibrosis confirmed by HRCT was observed in 42 subjects (70%), while PAH occurred in 4 patients (6.7%). Cardiac involvement was detected in 30 patients (50%), whereas renal involvement was not observed in any patient.

Antinuclear antibody (ANA) positivity was found in 85% of patients (*n* = 51), anticentromere antibody (ACA) positivity in 18.3% (*n* = 11), and anti-Scl-70 antibody positivity in 36.7% (*n* = 22). Anti-RNA polymerase antibody positivity was confirmed in 6.7% of patients (*n* = 4).

Among patients with SSc (*n* = 60), the prevalence of hand joint contractures was 36.7% (*n* = 22). 40% of patients (*n* = 24) had experienced fingertip ulcers at some point in their lives. Subcutaneous calcinosis was documented in 12 patients (20%), while shortened fingers were observed in 8 patients (13.3%). Telangiectasia was present in 56 patients (93.3%). The median mRSS was 7 (3.25; 9.75).

### Measurement properties of the Hungarian SSPRO questionnaire

#### Scaling properties

Floor and ceiling effects were examined using descriptive statistics. Fewer than 5% of respondents achieved the minimum (*n* = 1, 1.6%) or maximum (*n* = 0, 0%) possible SSPRO score, indicating the absence of relevant floor or ceiling effects.

### Structural validity

Structural validity was explored using principal component analysis. Based on the Kaiser criterion (eigenvalues > 1), a three-component solution was identified. The first component showed a strong eigenvalue (8.988) and accounted for 49.932% of the total variance, indicating a dominant underlying factor. The second and third components had substantially lower eigenvalues (1.661 and 1.520), explaining 9.228% and 8.445% of the variance, respectively. Together, these three components accounted for 67.605% of the total variance. Given the dominance of the first component and the relatively limited contribution of the additional components, these findings suggest a predominantly unidimensional structure with secondary underlying dimensions.

### Internal consistency

Internal consistency of the Hungarian SSPRO questionnaire was acceptable, with a Cronbach’s alpha coefficient of 0.789.

### Convergent validity

Convergent validity was assessed by examining correlations between SSPRO scores and comparator instruments measuring related constructs, using Spearman correlation analysis. In the overall SSc population, significant correlations were observed between the SSPRO questionnaire and HAQ-DI, SF-36, DLQI, Brief-SWAP, sHAQ, fatigue VAS, and questionnaires assessing skin thickness and thinness. Similar findings were obtained in patients with lcSSc. In the dcSSc subgroup, significant associations were found with HAQ-DI, SF-36, DLQI, fatigue VAS, and questionnaires evaluating skin thickness (Table 1).

Convergent validity was also analyzed in subgroups stratified by disease duration. Patients with disease duration shorter than four years (*n* = 10) were classified as the early group, while those with disease duration longer than four years formed the late group (*n* = 50). In the late group, SSPRO scores showed significant correlations with multiple instruments, including HAQ-DI, SF-36, DLQI, Brief-SWAP, sHAQ, fatigue VAS, and skin thickness/thinness assessments. In the early group, correlations were also observed with HAQ-DI, SF-36, and DLQI (Table 1).

**Table 1:** Assessment of convergent validity (Spearman Correlation Analysis)

	All patients (*n* = 60)		Limited cutaneous SSc (*n* = 29)		Diffuse cutaneous SSc (*n* = 31)		early SSc(*n* = 10)		late SSc (*n* = 50)	
	Rho	*p*	Rho	*p*	Rho	*p*	Rho	*p*	Rho	*p*
Health Assessment Questionnaire-Disability Index	**0.499**	**< 0.001**	**0.544**	**0.002**	**0.457**	**0.021**	**0.653**	**0.041**	**0.621**	**< 0.001**
SF-36-physical component	**-0.570**	**< 0.001**	**-0.663**	**< 0.001**	**-0.476**	**0.010**	**− 0.650**	**0.042**	**− 0.591**	**< 0.001**
SF-36-mental component	**-0.597**	**< 0.001**	**-0.769**	**< 0.001**	**-0.401**	**0.046**	**− 0.650**	**0.042**	**− 0.562**	**< 0.001**
Dermatology Life Quality Index	**0.754**	**< 0.001**	**0.767**	**< 0.001**	**0.760**	**< 0.001**	**0.724**	**0.018**	**0.759**	**< 0.001**
Brief-SWAP	**0.459**	**< 0.001**	**0.674**	**< 0.001**	0.221	0.286	0.396	0.257	**0.388**	**0.005**
sHAQ-Raynaud scale	0.274	0.045	0.312	0.099	0.215	0.301	0.113	0.756	**0.292**	**< 0.001**
sHAQ-fingertip ulcer scale	0.221	0.108	**0.294**	**0.121**	0.149	0.465	0.334	0.346	**0.292**	**0.039**
sHAQ-gastrointestinal involvement scale	**0.336**	**0.013**	**0.468**	**0.010**	0.189	0.363	-0.013	0.971	**0.387**	**0.005**
sHAQ-pulmonary involvement scale	**0.277**	**0.041**	**0.402**	**0.030**	0.110	0.577	0.112	0.758	0.261	0.067
Fatigue visual analog scale	**0.545**	**< 0.001**	**0.614**	**< 0.001**	**0.459**	**0.021**	0.619	0.056	**0.487**	**< 0.001**
Questionnaire Assessing Skin Thinness	**0.383**	**0.004**	0.354	0.059	0.369	0.068	− 0.101	0.781	**0.338**	**0.016**
Questionnaire Assessing Skin Thickness	**0.535**	**< 0.001**	**0.432**	**0.019**	**0.605**	**0.001**	0.529	0.116	**0.319**	**0.024**

SSc: systemic sclerosis; Brief-Swap: Satisfaction With Appearance Scale Brief-form; sHAQ: Scleroderma-specific Health Assessment Questionnaire.

**Fig. 1:**
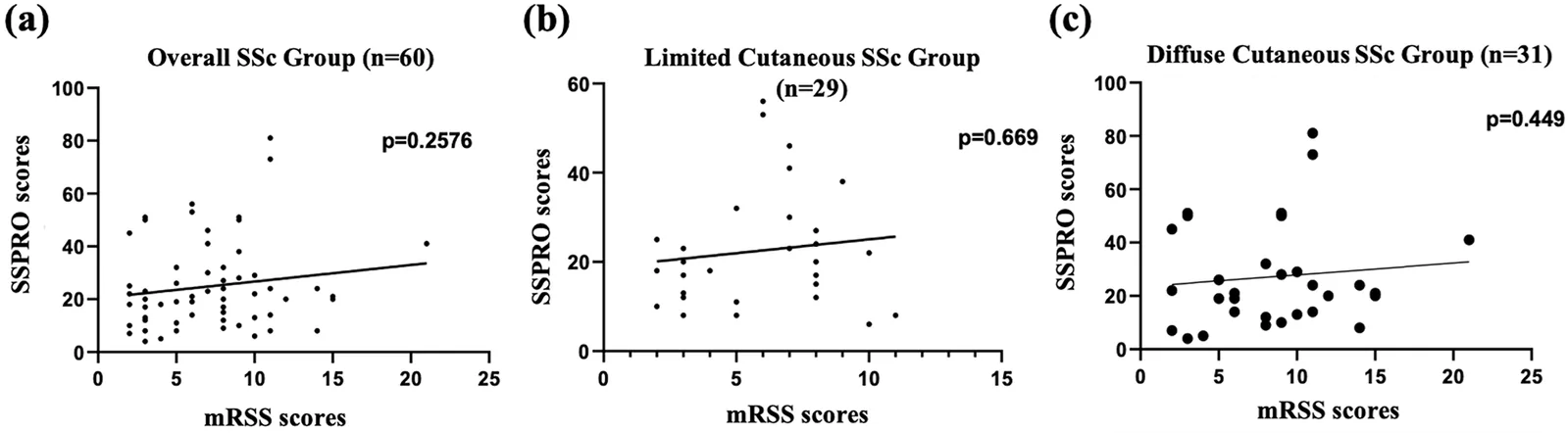
Correlation between SSPRO scores and mRSS scores: (**a**) Overall Systemic Sclerosis (SSc) Group; (**b**) Limited Cutaneous SSc Group; (**c**) Diffuse Cutaneous SSc Group

As part of the convergent validity analysis, we also assessed the association between the SSPRO total score and the mRSS. Spearman’s correlation analysis revealed no significant correlation between the two measures in the overall SSc population (Rho = 0.022, *p* = 0.258), nor within the lc SSc subgroup (Rho = 0.083, *p* = 0.669) or the dcSSc subgroup (Rho = 0.129, *p* = 0.499) (Fig. 1).

### Known-groups validity

**Fig. 2:**
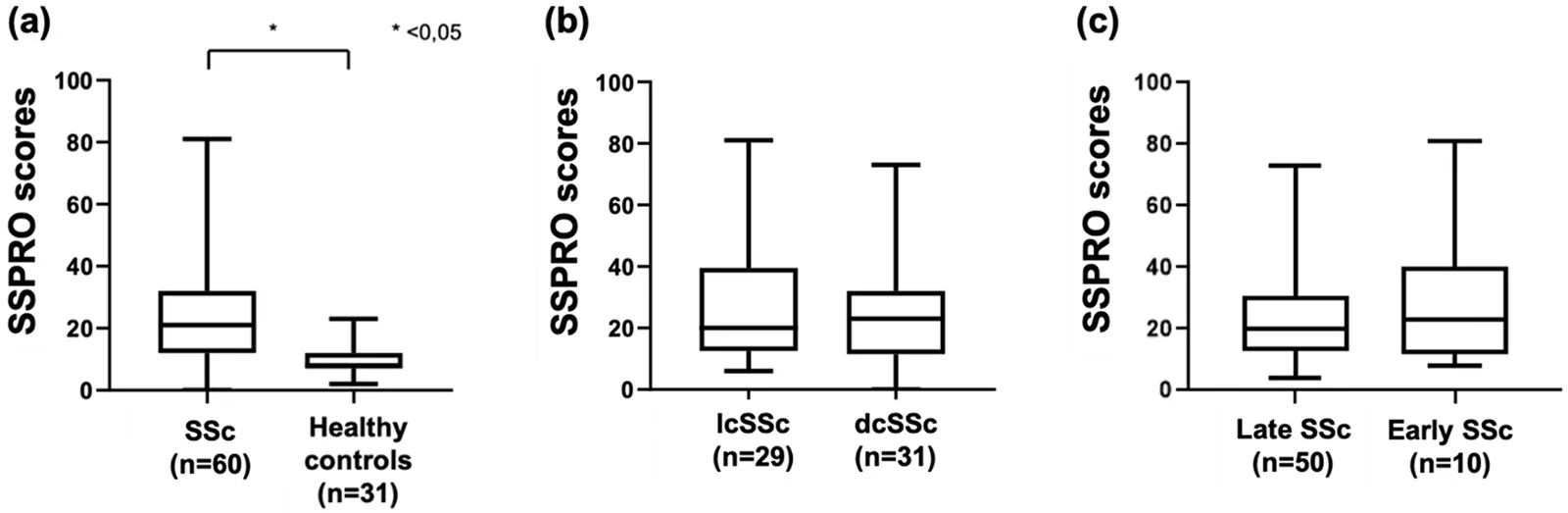
Known-groups Validity: (**a**) Patients with Systemic Sclerosis (SSc) and Healthy Controls; (**b**) Limited Cutaneous SSc (lcSSc) Group and Diffuse Cutaneous SSc (dcSSc) Group; (**c**) Late SSc Group and Early SSc Group

Known-groups validity was assessed using the Mann-Whitney U test to compare SSPRO scores between predefined groups. The questionnaire effectively differentiates between patients with SSc and healthy controls (Fig. 2). However, the questionnaire does not distinguish between lcSSc and dcSSc patients (Fig. 2). Furthermore, no significant differences in SSPRO scores were observed between early and late disease duration subgroups (Fig. 2).

### Reproducibility

To assess reproducibility, participants completed the questionnaire again on day 5 after the initial administration. The 5 day interval was selected based on routine clinical practice in our tertiary care center, aiming to minimize clinical change while avoiding recall bias. Test–retest reliability demonstrated excellent agreement, with an intraclass correlation coefficient (ICC(2,1), absolute agreement) of 0.894 (95% CI: 0.764–0.932).

## Discussion

The adaptation and validation of the SSPRO questionnaire into Hungarian can be considered successful.

Content validity of the Hungarian SSPRO was supported during the translation and cultural adaptation process, as the items were reviewed by an expert committee and pilot-tested with patients to ensure relevance, clarity, and comprehensibility.

Structural validity was explored using principal component analysis, which suggested a three-component solution with a clearly dominant first component explaining approximately 50% of the variance. This indicates that the Hungarian SSPRO functions primarily as a unidimensional instrument with secondary underlying dimensions. Notably, the original SSPRO validation study by Man et al. identified a multidimensional factor structure using exploratory factor analysis, with four components corresponding to physical effects, physical limitations, emotional effects, and social effects [20]. Therefore, the presence of multiple components in the Hungarian version is consistent with the conceptual framework of the original instrument. However, the presence of a component with a small number of items and the relatively limited sample size indicate that these findings should be interpreted cautiously. Larger multicenter studies are warranted to further clarify the dimensional structure using confirmatory approaches.

The convergent validity analysis found significant correlations between the SSPRO questionnaire results and the scores of the comparator questionnaires both in the overall SSc population and within the subgroups, which is consistent with the values reported in the English validation results [20] (Table 1). This indicates that participants who experienced a greater decline in quality of life due to skin involvement scored higher on the SSPRO, and this trend was consistent with the results of the previously validated comparator questionnaires [17, 18, 22–26]. The significant correlations suggest that the observed associations are unlikely to be due to chance, and that the SSPRO reliably reflects the extent of quality of life impairment caused by skin involvement [38].

SSPRO scores were associated with quality of life impairment in both early and established disease [39]. However, the markedly smaller sample size of the early disease subgroup (50 late-stage patients compared to 10 in the early-stage group) limits the strength of conclusions regarding differences across disease duration, and these findings should be interpreted with caution.

An important finding of our study is the lack of significant correlation between the SSPRO and the mRSS (Fig. 1). This indicates that the subjective burden of skin symptoms, as perceived and reported by patients, does not necessarily mirror the extent of skin thickening measured by clinicians. While the mRSS remains a key objective marker of skin fibrosis and disease activity, it captures only a limited facet of the patient experience. In contrast, the SSPRO reflects the functional, psychological, and social consequences of skin involvement, including discomfort, aesthetic concerns, frustration, and limitations in daily activities. These aspects often contribute more profoundly to quality of life impairment than can be inferred from objective skin scores alone. The divergence between mRSS and SSPRO therefore underscores the complementary role of patient-reported outcome measures in SSc, highlighting their importance for both clinical care and research, as they provide insights into disease impact that are not accessible through conventional physician-based assessments.

In contrast to the original validation study, the Hungarian SSPRO did not demonstrate significant differences between lcSSc and dcSSc subgroups [20]. This finding is likely multifactorial. First, the overall skin severity of the cohort was relatively mild, as reflected by the low median mRSS (7), which may have limited detectable clinical differences between subtypes. In addition, selection bias cannot be entirely excluded, although patients were enrolled consecutively; the majority of patients had long-standing disease, and at this stage, skin involvement often enters a more atrophic phase, which may result in lower modified Rodnan Skin Scores even in patients with diffuse cutaneous systemic sclerosis. Second, skin involvement of the hands and face, regions that have a disproportionate impact on daily functioning, communication, and social interaction, is common to both lcSSc and dcSSc and may represent a major driver of patient-perceived disease burden captured by the SSPRO [3]. Consequently, subjective quality-of-life impairment related to skin involvement may be similar across subtypes despite differences in overall skin extent. Additionally, the modest sample size limits statistical power for subgroup analyses, and future studies focusing on patients with early diffuse disease and higher skin scores would be valuable to further evaluate the discriminatory capacity of the SSPRO.

Moreover, the Hungarian adaptation offers unique and forward-looking contributions. To our knowledge, this is the first SSPRO validation study to include a healthy control group (*n* = 31), allowing a clear assessment of known-groups validity. While the English and Turkish studies included only patients with SSc, the Hungarian version of the SSPRO was able to clearly distinguish the responses of SSc patients from those of the healthy population (*p* < 0.001). This approach not only reinforces the sensitivity of the questionnaire but also contributes to broadening its clinical applicability [20, 40].

The integration of the SSPRO questionnaire into routine clinical care could represent a significant advancement in the comprehensive assessment of quality of life in patients with SSc. The questionnaire is quick to complete, easy to interpret, and fits well into the workflow of outpatient care.

In clinical practice, the use of the SSPRO allows for the documentation of patients’ subjective experiences of skin symptoms, complementing the objective mRSS assessment. Regular use of the questionnaire may assist in monitoring treatment effectiveness, particularly when therapies targeting skin involvement are applied. By capturing patient-reported physical, emotional, and social consequences of skin involvement, the SSPRO may help identify areas where supportive interventions are needed. Based on SSPRO results, referral to multidisciplinary care, including psychological or physiotherapeutic support, may be considered to address specific patient-reported needs.

The limitations of the present study include the relatively small sample size (*n* = 60) and the single center study design. Although the cohort was representative of the Hungarian SSc population, the limited number of participants, particularly within subgroup analyses and in the control group, constrains statistical power and generalizability. Accordingly, there remains an unmet need for larger multicenter studies to further evaluate the measurement properties and dimensional structure of the Hungarian version of the SSPRO questionnaire.

In summary, the adaptation and validation of the Scleroderma Skin Patient-Reported Outcome (SSPRO) questionnaire into Hungarian can be considered successful. The questionnaire is a quick and simple instrument with acceptable measurement properties for assessing skin-related quality of life impairment in patients with SSc.

## Supplementary Information

Below is the link to the electronic supplementary material.

Supplementary Material 1

## Data Availability

The datasets generated and analyzed during the current study are available from the corresponding author on reasonable request.

## Abbreviations

SSc: Systemic sclerosis
PAH: Pulmonary arterial hypertension
PROs: Patient–reported outcomes
PROMs: Patient–reported outcome measures
HAQ–DI: Health Assessment Questionnaire–Disability Index
sHAQ: scleroderma Health Assessment Questionnaire
GIT 2.0: UCLA Scleroderma Gastrointestinal Tract Instrument
SSPRO: Scleroderma Skin Patient–Reported Outcome
dcSSc: diffuse cutaneous SSc
lcSSc: limited cutaneous SSc
SF: 36Short Form–36 Health Survey
DLQI: Dermatology Life Quality Index
Brief–SWAP: Satisfaction With Appearance Scale–Brief form
VAS: Visual Analogue Scale
mRSS: modified Rodnan Skin Score
ILD: Interstitial lung disease
HRCT: High–resolution computed tomography
RHC: Right heart catheterization
ANA: Antinuclear antibody
ACA: Anticentromere antibody
ICC: Intraclass correlation coefficient

## References

[1] Gabrielli A, Avvedimento EV, Krieg T, Scleroderma (2009) N Engl J Med 360(19):1989–2003. 10.1056/NEJMra0806188 19420368

[2] Allanore Y, Simms R, Distler O et al (2015) Systemic sclerosis. Nat Rev Dis Primers 1:15002 Published 2015 Apr 23. 10.1038/nrdp.2015.2 27189141

[3] van Leeuwen NM, Liem SIE, Maurits MP et al (2021) Disease progression in systemic sclerosis. Rheumatology (Oxford) 60(3):1565–1567. 10.1093/rheumatology/keaa911 PMC7937017 33404661

[4] Maciejewska M, Sikora M, Maciejewski C, Alda-Malicka R, Czuwara J, Rudnicka L (2022) Raynaud’s Phenomenon with Focus on Systemic Sclerosis. J Clin Med 11(9):2490 Published 2022 Apr 28. 10.3390/jcm11092490 PMC9105786 35566614

[5] Siqueira VS, Helbingen MFS, Medeiros-Ribeiro AC et al (2022) Predictors of progression to systemic sclerosis: analysis of very early diagnosis of systemic sclerosis in a large single-centre cohort. Rheumatology (Oxford) 61(9):3686–3692. 10.1093/rheumatology/keac006 35020814

[6] Yin H, Lin W, Lu L (2023) Puffy fingers and abnormal nail fold capillaries. CMAJ 195(39):E1350. 10.1503/cmaj.230834 PMC10610952 37816528

[7] Ross L, Maltez N, Hughes M et al (2023) Systemic pharmacological treatment of digital ulcers in systemic sclerosis: a systematic literature review. Rheumatology (Oxford) 62(12):3785–3800. 10.1093/rheumatology/kead289 PMC10691932 37335850

[8] Halachmi S, Gabari O, Cohen S, Koren R, Amitai DB, Lapidoth M (2014) Telangiectasis in CREST syndrome and systemic sclerosis: correlation of clinical and pathological features with response to pulsed dye laser treatment. Lasers Med Sci 29(1):137–140. 10.1007/s10103-013-1298-1 23494102

[9] Lescoat A, Huang S, Carreira PE et al (2023) Cutaneous Manifestations, Clinical Characteristics, and Prognosis of Patients With Systemic Sclerosis Sine Scleroderma: Data From the International EUSTAR Database. JAMA Dermatol 159(8):837–847. 10.1001/jamadermatol.2023.1729 PMC10308295 37378994

[10] Filipánits K, Nagy G, Varjú C, Czirják L, Minier T (2024) Microstomia is associated with functional impairment and is a poor prognostic factor in systemic sclerosis - a single center observational study with survival analysis. BMC Oral Health 24(1):1390. 10.1186/s12903-024-05178-6. Published 2024 Nov 15 PMC11568575 39548478

[11] Hudson M, Thombs BD, Steele R et al (2009) Health-related quality of life in systemic sclerosis: a systematic review. Arthritis Rheum 61(8):1112–1120. 10.1002/art.24676 19644906

[12] Weldring T, Smith SM (2013) Patient-Reported Outcomes (PROs) and Patient-Reported Outcome Measures (PROMs). Health Serv Insights 6:61–68 Published 2013 Aug 4. 10.4137/HSI.S11093 PMC4089835 25114561

[13] Black N (2013) Patient reported outcome measures could help transform healthcare. BMJ 346:f167 Published 2013 Jan 28. 10.1136/bmj.f167 23358487

[14] Deshpande PR, Rajan S, Sudeepthi BL, Abdul Nazir CP (2011) Patient-reported outcomes: A new era in clinical research. Perspect Clin Res 2(4):137–144. 10.4103/2229-3485.86879 PMC3227331 22145124

[15] Khanna D, Merkel PA (2007) Outcome measures in systemic sclerosis: an update on instruments and current research. Curr Rheumatol Rep 9(2):151–157. 10.1007/s11926-007-0010-5 17502046

[16] Boers M, Kirwan JR, Wells G et al (2014) Developing core outcome measurement sets for clinical trials: OMERACT filter 2.0. J Clin Epidemiol 67(7):745–753. 10.1016/j.jclinepi.2013.11.013 24582946

[17] Steen VD, Medsger TA Jr (1997) The value of the Health Assessment Questionnaire and special patient-generated scales to demonstrate change in systemic sclerosis patients over time. Arthritis Rheum 40(11):1984–1991. 10.1002/art.1780401110 9365087

[18] Johnson SR, Hawker GA, Davis AM (2005) The health assessment questionnaire disability index and scleroderma health assessment questionnaire in scleroderma trials: an evaluation of their measurement properties. Arthritis Rheum 53(2):256–262. 10.1002/art.21084 15818719

[19] Baron M, Hudson M, Steele R, Lo E, Canadian Scleroderma Research Group (2011) Validation of the UCLA Scleroderma Clinica Trial Gastrointestinal Tract Instrument version 2.0 for systemic sclerosis. J Rheumatol 38(9):1925–1930. 10.3899/jrheum.110060 21724704

[20] Man A, Correa JK, Ziemek J, Simms RW, Felson DT, Lafyatis R (2017) Development and validation of a patient-reported outcome instrument for skin involvement in patients with systemic sclerosis. Ann Rheum Dis 76(8):1374–1380. 10.1136/annrheumdis-2016-210534 28213563

[21] van den Hoogen F, Khanna D, Fransen J et al (2013) 2013 classification criteria for systemic sclerosis: an American college of rheumatology/European league against rheumatism collaborative initiative. Ann Rheum Dis 72(11):1747–1755. 10.1136/annrheumdis-2013-204424 24092682

[22] Ware JE Jr, Sherbourne CD (1992) The MOS 36-item short-form health survey (SF-36). I. Conceptual framework and item selection. Med Care 30(6):473–483 1593914

[23] Finlay AY, Khan GK (1994) Dermatology Life Quality Index (DLQI)--a simple practical measure for routine clinical use. Clin Exp Dermatol 19(3):210–216. 10.1111/j.1365-2230.1994.tb01167.x 8033378

[24] Jewett LR, Hudson M, Haythornthwaite JA et al (2010) Development and validation of the brief-satisfaction with appearance scale for systemic sclerosis. Arthritis Care Res (Hoboken) 62(12):1779–1786. 10.1002/acr.20307 20665750

[25] Lee KA, Hicks G, Nino-Murcia G (1991) Validity and reliability of a scale to assess fatigue. Psychiatry Res 36(3):291–298. 10.1016/0165-1781(91)90027-m 2062970

[26] Nagy Z, Bálint Z, Farkas H et al (2009) Establishment and partial validation of a patient skin self-assessment questionnaire in systemic sclerosis. Rheumatology (Oxford) 48(3):309–314. 10.1093/rheumatology/ken490 19181657

[27] Nihtyanova SI, Denton CP (2010) Autoantibodies as predictive tools in systemic sclerosis. Nat Rev Rheumatol 6(2):112–116. 10.1038/nrrheum.2009.238 20125179

[28] Allanore Y, Meune C, Kahan A (2008) Systemic sclerosis and cardiac dysfunction: evolving concepts and diagnostic methodologies. Curr Opin Rheumatol 20(6):697–702. 10.1097/BOR.0b013e328313bcf1 18946331

[29] Jouvray M, Launay D, Dubucquoi S et al (2018) Whole-Body Distribution and Clinical Association of Telangiectases in Systemic Sclerosis. JAMA Dermatol 154(7):796–805. 10.1001/jamadermatol.2018.0916 PMC6128493 29799952

[30] Khanna D, Furst DE, Clements PJ et al (2017) Standardization of the modified Rodnan skin score for use in clinical trials of systemic sclerosis. J Scleroderma Relat Disord 2(1):11–18. 10.5301/jsrd.5000231 PMC5431585 28516167

[31] Distler O, Brown KK, Distler JHW et al (2017) Design of a randomised, placebo-controlled clinical trial of nintedanib in patients with systemic sclerosis-associated interstitial lung disease (SENSCIS™). Clin Exp Rheumatol 35(Suppl 106):75–81 28664834

[32] Humbert M, Kovacs G, Hoeper MM et al (2023) 2022 ESC/ERS Guidelines for the diagnosis and treatment of pulmonary hypertension. Eur Respir J 61(1):2200879 Published 2023 Jan 6. 10.1183/13993003.00879-2022 36028254

[33] Ross L, Prior D, Proudman S, Vacca A, Baron M, Nikpour M (2019) Defining primary systemic sclerosis heart involvement: A scoping literature review. Semin Arthritis Rheum 48(5):874–887. 10.1016/j.semarthrit.2018.07.008 30170705

[34] Denton CP, Khanna D (2017) Systemic sclerosis. Lancet 390(10103):1685–1699. 10.1016/S0140-6736(17)30933-9 28413064

[35] Walker UA, Tyndall A, Czirják L et al (2007) Clinical risk assessment of organ manifestations in systemic sclerosis: a report from the EULAR Scleroderma Trials And Research group database. Ann Rheum Dis 66(6):754–763. 10.1136/ard.2006.062901 PMC1954657 17234652

[36] Mokkink LB, Terwee CB, Patrick DL et al (2010) The COSMIN checklist for assessing the methodological quality of studies on measurement properties of health status measurement instruments: an international Delphi study. Qual Life Res 19(4):539–549. 10.1007/s11136-010-9606-8 PMC2852520 20169472

[37] Beaton DE, Bombardier C, Guillemin F, Ferraz MB (2000) Guidelines for the process of cross-cultural adaptation of self-report measures. Spine (Phila Pa 1976) 25(24):3186–3191. 10.1097/00007632-200012150-00014 11124735

[38] Nachar N (2008) The Mann-Whitney U: a test for assessing whether two independent samples come from the same distribution. tutorials in quantitative methods for psychology. 10.20982/tqmp.04.1.p013.

[39] Almeida C, Almeida I, Vasconcelos C (2015) Quality of life in systemic sclerosis. Autoimmun Rev 14(12):1087–1096. 10.1016/j.autrev.2015.07.012 26212726

[40] Sari F, Bazancir Apaydin Z, Apaydin H et al (2023) Reliability and validity of the Turkish version of Scleroderma Skin Patient-Reported Outcome in patients with systemic sclerosis. Arch Rheumatol 39(1):52–59 Published 2023 Jun 14. 10.46497/ArchRheumatol.2023.10183 PMC11104756 38774706

