# Supplementary material for: Adaptation and validation of the scleroderma skin patient-reported outcome questionnaire into Hungarian
Source: J Patient Rep Outcomes. 2026 Jul 14;10:182. doi: 10.1186/s41687-026-01147-5 (PMC13646231; doi:10.1186/s41687-026-01147-5)
Supplement: Supplementary file 1 [file 41687_2026_1147_MOESM1_ESM.docx]

|  | **0** | **1** | **2** | **3** | **4** | **5** | **6** |
| --- | --- | --- | --- | --- | --- | --- | --- |
|  | **Egyáltalán nem** |  |  |  |  |  | **Teljes mértékben** |
| **Mennyire érezte feszesnek a bőrét?** |  |  |  |  |  |  |  |
| **Mennyire volt száraz a bőre?** |  |  |  |  |  |  |  |
| **Mennyire volt fájdalmas a bőre?** |  |  |  |  |  |  |  |
| **Mennyire színeződött el a bőre?** |  |  |  |  |  |  |  |
| **Mennyire viszketett a bőre?** |  |  |  |  |  |  |  |
| **Mennyire volt magabiztos a bőre miatt?** |  |  |  |  |  |  |  |
| **Mennyire aggódott a bőre miatt?** |  |  |  |  |  |  |  |
| **Mennyire volt elkeseredett a bőre miatt?** |  |  |  |  |  |  |  |
| **Mennyire érezte úgy a bőre miatt, mintha nem önmaga lenne?** |  |  |  |  |  |  |  |
| **Mennyire volt frusztrált a bőre miatt?** |  |  |  |  |  |  |  |
| **Mennyire érezte úgy, hogy nem képes befolyásolni a bőre állapotát?** |  |  |  |  |  |  |  |
| **Mekkora nehézséget okozott Önnek, hogy kézzel csináljon dolgokat a bőr feszülés miatt?** |  |  |  |  |  |  |  |
| **Mennyire volt nehéz kinyitni vagy becsukni a száját a bőr feszülése miatt?** |  |  |  |  |  |  |  |
| **A bőr feszessége miatt mennyire esett nehezére a testrészeit mozgatni?** |  |  |  |  |  |  |  |
| **Mennyire befolyásolta a bőre állapota a mindennapi tevékenységeit (pl. munka, tanulás, szabadidős tevékenységek)?** |  |  |  |  |  |  |  |
| **Mennyire akadályozta meg bőre abban, hogy társaságba menjen?** |  |  |  |  |  |  |  |
| **Mennyire befolyásolta Önt a bőre az emberekkel való interakcióját?** |  |  |  |  |  |  |  |
| **Mennyire befolyásolta a bőre abban, hogy milyen ruhát visel?** |  |  |  |  |  |  |  |

**Appendix A: Hungarian version of the Scleroderma Skin Patient-Reported Outcome Questionnaire**
